# Circulating microRNAs as promising diagnostic biomarkers for hepatocellular carcinoma: a systematic review and meta-analysis

**DOI:** 10.3389/fmolb.2024.1353547

**Published:** 2024-05-14

**Authors:** Ermiyas Alemayehu, Alebachew Fasil, Hussen Ebrahim, Zewudu Mulatie, Getachew Mesfin Bambo, Alemu Gedefie, Mulugeta Teshome, Abebaw Worede, Melaku Ashagrie Belete

**Affiliations:** ^1^ Department of Medical Laboratory Sciences, College of Medicine and Health Sciences, Wollo University, Dessie, Ethiopia; ^2^ Department of Clinical Chemistry, School of Biomedical and Laboratory Sciences, College of Medicine and Health Sciences, University of Gondar, Gondar, Ethiopia; ^3^ Department of Medical Laboratory Science, College of Health Sciences, Mizan-Tepi University, Mizan Aman, Ethiopia; ^4^ Department of Medical Laboratory Science, Dessie Health Science College, Dessie, Ethiopia

**Keywords:** miRNAs, non-coding RNAs, diagnostic biomarkers, hepatocellular carcinoma, HCC, liver cancer, meta-analysis

## Abstract

**Introduction:** Hepatocellular carcinoma (HCC), the most common type of liver cancer, is a major global health problem, ranking as the third leading cause of cancer-related death worldwide. Early identification and diagnosis of HCC requires the discovery of reliable biomarkers. Therefore, the study aimed to assess the diagnostic accuracy of miRNAs for HCC. The protocol was registered on PROSPERO website with the registration number CRD42023417494.

**Method:** A literature search was conducted in PubMed, Scopus, Embase, Wiley Online Library, and Science Direct databases to identify pertinent articles published between 2018 and 30 July 2023. Stata 17.0 software was employed to determine the pooled sensitivity, specificity, positive likelihood ratio (PLR), negative likelihood ratio (NLR), diagnostic ratio (DOR), and area under the curve (AUC) for evaluating the accuracy of miRNAs in diagnosing HCC. The assessment of heterogeneity among studies involved the use of the Cochran-Q test and I^2^ statistic tests. Due to the observed significant heterogeneity, the random-effect model was chosen. Subgroup analysis and meta-regression analysis were also undertaken to explore potential sources contributing to heterogeneity. Deeks’ funnel plot was used to assess publication bias. In addition, Fagan’s nomogram and likelihood ratio scattergram were utilized to assess the clinical validity of miRNAs for HCC.

**Result:** Twenty-four articles were included, involving 1,668 individuals diagnosed with HCC and 1,236 healthy individuals. The findings revealed pooled sensitivity of 0.84 (95% CI: 0.80–0.88), specificity of 0.81 (95% CI: 0.77–0.84), PLR of 4.36 (95% CI: 3.59–5.30), NLR of 0.19 (95% CI: 0.15–0.25), DOR of 22.47 (95% CI: 14.47–32.64), and an AUC of 0.89 (95% CI: 0.86–0.91) for the diagnosis of HCC using miRNAs. Furthermore, results from the subgroup analysis demonstrated that superior diagnostic performance was observed when utilizing plasma miRNAs, a large sample size (≥100), and miRNA panels.

**Conclusion:** Hence, circulating miRNAs demonstrate substantial diagnostic utility for HCC and can serve as effective non-invasive biomarkers for the condition. Additionally, miRNA panels, miRNAs derived from plasma, and miRNAs evaluated in larger sample sizes (≥100) demonstrate enhanced diagnostic efficacy for HCC diagnosis. Nevertheless, a large pool of prospective studies and multi-center research will be required to confirm our findings in the near future.

## Introduction

Hepatocellular carcinoma (HCC) stands as a formidable global health challenge, representing the most common primary malignancy of the liver accounting for approximately 90% of cases (Singal et al., 2023). It is ranked as the seventh most frequently occurring cancer, and the third leading cause of cancer-related mortality worldwide with over 800,000 deaths annually (Arnold et al., 2020; Sung et al., 2021). The World Health Organization (WHO) forecasts that the annual death rate from HCC will spike to more than 1 million individuals by the year 2030 (World Health Organization, 2020).

Despite therapeutic strategies are nowadays advanced, the prognosis for HCC remains suboptimal mainly due to late-stage diagnosis and limited therapeutic options (Guan et al., 2021). This is evidenced by poor overall prognosis worldwide, with global age-standardized incidence and mortality at 9.5 and 8.7 per 100,000 person-years, respectively (Rumgay et al., 2022). Moreover, mainly attributable to the absence of obvious clinical symptoms in patients with early HCC, early diagnosis of HCC is still very difficult and insufficient.

Currently, HCC screening primarily relies on cross-sectional imaging such as magnetic resonance imaging (MRI), computed tomography (CT) scanning, ultrasonography (US), contrast-enhanced ultrasound (CEUS), and some tumor markers, particularly α-fetoprotein (AFP) (Frenette et al., 2019). However, such screening techniques still have major shortcomings in detecting early onset HCC (Sherman, 2014). For instance, low (more than 40%) AFP positivity rate frequently observed for early liver cancer, and as a result of which the American Association for the Study of Liver Diseases (AASLD) and the European Association for the Study of the Liver (EASL) (European Association for the Study of the LiverEuropean Organisation For and Research And Treatment Of Cancer, 2012) have excluded AFP as a diagnostic marker for HCC (Bae, 2012; Parra et al., 2023). Furthermore, pathological tests are invasive and can lead to complications (Martins-Filho and Alves, 2019). Therefore, there is an urgent need for robust, less invasive, and reliable biomarkers that can facilitate early diagnosis, prognosis, and therapeutic monitoring in HCC patients.

Recently, there has been a growing interest in the role of microRNAs (miRNAs) as diagnostic biomarkers for numerous cancers, including HCC. MicroRNAs are a class of small (approximately 19–24 nucleotides), non-coding RNAs that play critical roles in the post-transcriptional regulation of gene expression (Armand-Labit and Pradines, 2017). Their dysregulation has been implicated in the initiation and progression or pathogenesis of different diseases, making them promising targets for biomarker discovery (Valihrach et al., 2020; Kim and Croce, 2023). The stability of circulating miRNAs in body fluids such as blood, plasma serum and urine, and their non-invasive potential make them preferable diagnostic biomarkers unlike tissue-based biomarkers (Wang et al., 2012). This is particularly vital in HCC, where tissue sampling may become invasive and risky, and pose challenges. The ability of circulating miRNAs to indicate the molecular changes in the cancer microenvironment embraces their remarkable utilization in clinical settings (Gramantieri et al., 2021).

Several studies have emphasized the altered expression patterns and dysregulation of specific circulating miRNAs in the development, progression and metastasis of HCC, signifying their utility as promising diagnostic biomarkers (Jin et al., 2019; Fang et al., 2022; Lv and Sun, 2023), but comprehensive and up-to-date evidence-based data is still lacking. In this systematic review and meta-analysis, we aimed to comprehensively evaluate the current state of evidence regarding the diagnostic potential of circulating miRNAs as reliable, non-invasive and clinically applicable biomarkers for the detection of HCC intending to provide an input in addressing the unmet needs in early detection of HCC.

The findings hold significant relevance and applicability in the clinical setting, particularly for improving the early detection of HCC, which is crucial for enhancing patient outcomes, given the disease is often diagnosed at advanced stages when treatment options are limited. Furthermore, the non-invasive nature of circulating miRNA testing makes it particularly appealing for widespread clinical use. Additionally, the clinical utility of circulating miRNAs in HCC diagnosis is underscored by their potential integration into routine screening or diagnostic protocols. By incorporating these biomarkers into existing diagnostic algorithms, the accuracy and effectiveness of HCC detection could be enhanced, ultimately leading to improved patient management and outcomes.

## Methods and materials

### Registration

This research follows the guidelines outlined in the Preferred Reporting Items for Systematic Reviews and Meta-analysis (PRISMA) statement (Page et al., 2021). The study protocol was registered in the Prospective Register of Systematic Reviews (PROSPERO) under the registration identifier CRD42023417494.

### Search strategy and data sources

Two independent researchers (EA and MAB) carried out a comprehensive literature search to collect studies that assessed the diagnostic value of circulating miRNAs for HCC. Various electronic bibliographic databases, including PubMed, Scopus, Embase, Wiley Online Library, and Science Direct, were utilized for this purpose. Additionally, a direct search on Google was performed to identify any relevant studies that might have been omitted during the electronic database searches by checking the bibliographies of the identified studies. The final search was conducted on 30 July 2023. The search strategy incorporated Medical Subject Heading (MeSH) terms and keywords, such as “Serum” OR “plasma” OR “circulating” AND “miRNAs” OR “microRNAs” OR “miRNA” OR “microRNA” OR “miR” AND “diagnosis” AND “hepatocellular carcinoma” OR “HCC”. The detailed search strategy is available in the Supplementary Table S1.

### Inclusion and exclusion criteria

This review considered specific types of studies, namely, observational studies (including case-control, cross-sectional, and cohort studies) published in peer-reviewed journals since 2018, which explored the utility of circulating miRNAs as a diagnostic tool for distinguishing HCC patients from healthy individuals and examined miRNAs in plasma or serum. In addition, the studies had to provide essential data such as sensitivity, specificity, and sample sizes, enabling the calculation of key diagnostic metrics like true positives (TP), false positives (FP), false negatives (FN), and true negatives (TN). On the other hand, the review excluded review articles, case reports, narrative reviews, conference abstracts, editorials, commentaries, letters to the editor, author replies, studies that did not involve human subjects, and studies lacking the necessary data to calculate TP, FP, TN, and FN. These inclusion and exclusion criteria were employed to guide the selection of studies for the review.

### Study selection and data extraction

The studies gathered from the databases mentioned earlier, as well as from direct google search, were imported into EndNote 20 software to identify and eliminate duplicate entries. Subsequently, a thorough screening process was carried out for each selected paper, involving the evaluation of the title, abstract, and full text by two independent reviewers (EA and AF), in accordance with the predetermined eligibility criteria. In cases where discrepancies or disagreements arose between the two reviewers, a discussion took place, and a third reviewer (AG) was involved as needed to make the final determinations regarding which articles would be included in the review.

The selected papers underwent a thorough assessment, during which essential information was collected and organized into an extraction table using Microsoft Office Excel software. This process involved identifying key details, such as the first author, publication year, country of the study, extracted miRNAs, miRNA expression, type of specimen used, internal reference control, sample sizes for both HCC patients and healthy individuals, diagnostic methods, and cut-off values. Additionally, diagnostic parameters including sensitivity, specificity, and the area under the curve (AUC) were extracted by two independent investigators (AW and HE). To ensure accuracy and consistency, the findings were meticulously cross-checked by the two reviewers, and any discrepancies between the data extractors were resolved through discussion and consensus with the involvement of a third reviewer (GMB), thus verifying the integrity of the collected data.

### Quality assessment

The quality of the included studies was assessed using the modified Quality Assessment of Diagnostic Accuracy Studies-2 (QUADAS-2) tool (Freeman et al., 2017), which was carried out with the assistance of Review Manager 5.4 software. Two independent investigators (ZM and EA) conducted this assessment. The QUADAS-2 tool comprises four domains, namely, patient selection, index test, reference standard, and flow and timing. It entailed evaluating the clinical applicability of selected patients, the performance of the index test, and the standard of the reference. The risk of bias was categorized as high, low, or unclear based on this evaluation.

### Data analysis

The data analysis was performed using Stata version 17.0 software. To assess the heterogeneity among the studies, Cochrane Q test and I^2^ statistics were employed. Considerable heterogeneity was identified when the I^2^ test statistics values exceeded 50% and the *p*-value was less than 0.05 (Higgins et al., 2003). The available data were transformed into diagnostic parameters, including TP, FP, FN, and TN. These parameters were used to calculate the pooled sensitivity, specificity, positive likelihood ratio (PLR), negative likelihood ratio (NLR), diagnostic odds ratio (DOR), and area under the curve (AUC) using a random-effects model (Jackson et al., 2010). The AUC and DOR from the summary receiver characteristic curve (SROC) were used to evaluate the overall diagnostic accuracy of circulating miRNAs in diagnosing HCC. The existence of a threshold effect was established through an analysis of the Spearman correlation coefficient and visual inspection of the SROC curve. A *p*-value of less than 0.05 derived from the Spearman correlation coefficient, coupled with the presence of the characteristic “shoulder-arm” shape in the SROC curve, indicated the presence of a threshold effect. Subgroup analyses and meta-regression analyses were conducted to investigate the primary sources of heterogeneity. Deeks’ funnel plot asymmetry was employed to assess the presence of publication bias, where a *p*-value greater than 0.10 indicated the absence of publication bias. Moreover, sensitivity analysis was performed to assess the robustness and reliability of the results. Additionally, the Fagan plot and likelihood ratio scattergram were used to evaluate the clinical utility of miRNAs in distinguishing HCC patients from healthy individuals.

## Results

### Study selection process

**FIGURE 1:**
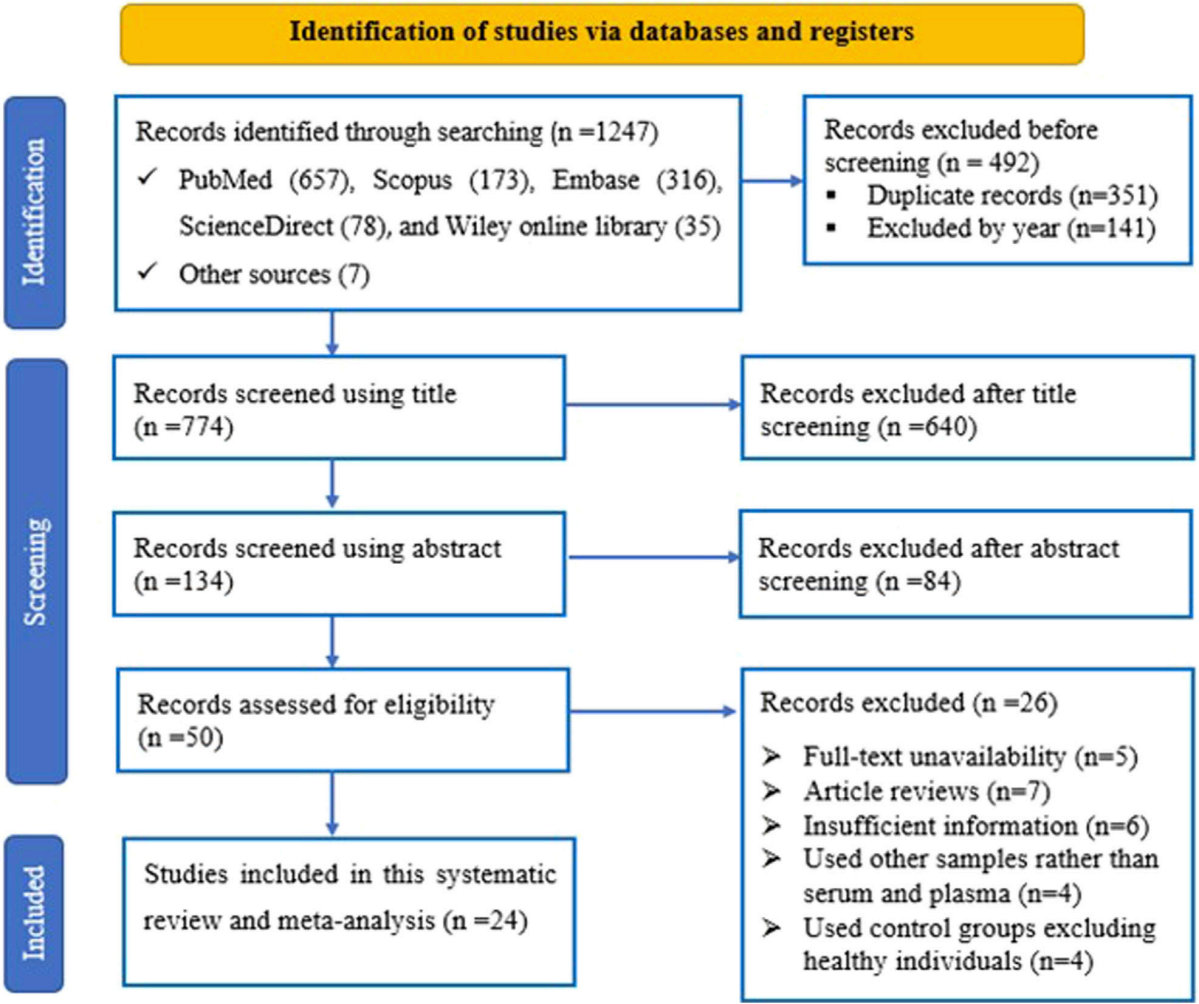
The PRISMA flow diagram illustrates the flow of study selection, detailing the number of studies identified, included, and excluded at each stage of the selection process according to predefined criteria.

A total of 1,266 records were retrieved from various online databases such as PubMed, Scopus, Embase, ScienceDirect, and Wiley library. Initially, 351 duplicated studies were removed, followed by excluding 141 articles based on their publication year. Subsequently, 724 studies including irrelevant ones like reviews, conference proceedings, and commentaries were excluded after reviewing their titles and abstracts. Then, 50 full-text articles were thoroughly assessed against specific criteria, resulting in the exclusion of 26 studies for various reasons. Ultimately, after this rigorous process, 50 studies from 24 articles were deemed suitable and met the criteria for inclusion in the meta-analysis. The visual representation outlining our screening process can be found in Figure 1.

### Characteristics, and quality assessment of included studies

Table 1 presents the fundamental characteristics of the literature encompassed in this analysis. The table includes data from 24 papers, which collectively involved 1,668 individuals diagnosed with HCC and 1,236 healthy individuals. These papers were published between 2018 and 2023, with thirteen originating from China (Xu L. et al., 2018; Lv et al., 2018; Chen and Wang, 2019; Han et al., 2019; Ning et al., 2019; Zhang et al., 2019; Cao and Wang, 2020; Wu et al., 2020; Zeng et al., 2020; Chen et al., 2021; Zhao et al., 2021; Fang et al., 2022; Wu et al., 2022), eight from Egypt (Shaheen et al., 2018; El Mahdy et al., 2019; Elmougy et al., 2019; Aly et al., 2020; Elhendawy et al., 2020; Wahb et al., 2021; Elfert et al., 2022; Youssef et al., 2022), one from India (Yousuf et al., 2022), one from Germany (Loosen et al., 2021), and one from Italy (Moshiri et al., 2018). In terms of sample types, eighteen studies utilized serum samples, while six studies used plasma samples for miRNA extraction. Methods for determining miRNA expression varied among the studies: one utilized next-generation sequencing, one used quantitative PCR, one employed droplet digital PCR, while the remaining twenty-two studies utilized quantitative real-time PCR. Regarding miRNA profiling, 44 studies reported on single miRNAs, while 6 studies focused on miRNAs panels. Among the findings, twenty studies reported an upregulation in miRNA expression, whereas twenty-four studies reported a downregulation.

**TABLE 1:** Intervening characteristics of the included studies.

Authors	Year	County	miRNAs	Expression	Specimen	Method	References	Participants				Cut-off	Sen (%)	Spe (%)	AUC
								Case	No	Control	No				
Xu et al. (Xu et al., 2018a)	2018	China	miR-125b	Down	Serum	qRT-PCR	U6	HBV-HCC	100	HC	100	2.45	83	96	0.94
Lv et al. (Lv et al., 2018)	2018	China	miR-21	Up	Serum	qRT-PCR	U6	HCC	75	HC	80	NA	69.1	78.3	0.771
Lv et al. (Lv et al., 2018)	2018	China	miR-214	Down	Serum	qRT-PCR	U6	HCC	75	HC	80	NA	79.3	69.6	0.733
Lv et al. (Lv et al., 2018)	2018	China	miR-15b	Up	Serum	qRT-PCR	U6	HCC	75	HC	80	NA	89.7	63.1	0.809
Lv et al. (Lv et al., 2018)	2018	China	miR-21, 214, 15b	NA	Serum	qRT-PCR	U6	HCC	75	HC	80	NA	80.3	87.0	0.887
Han et al. (Han et al., 2019)	2019	China	miR-148a	Down	Plasma	qRT-PCR	U6 snRNA	HCC	155	HC	95	8.12	97.9	92.9	0.980
Zhang et al. (Zhang et al., 2019)	2019	China	miR‐130b	Up	Serum	qRT-PCR	U6	HCC	46	HC	55	2.525	82.20	73.72	0.725
Zhang et al. (Zhang et al., 2019)	2019	China	miR‐21	Up	Serum	qRT-PCR	U6	HCC	46	HC	55	2.312	81.29	75.74	0.795
Zhang et al. (Zhang et al., 2019)	2019	China	miR‐21, 130b	Up	Serum	qRT-PCR	U6	HCC	46	HC	55	NA	92.16	77.51	0.832
Ning et al. (Ning et al., 2019)	2019	China	miR-155	Up	Serum	qRT-PCR	U6	HCC	30	HC	30	NA	93.3	70.0	0.840
Ning et al. (Ning et al., 2019)	2019	China	miR-96	Up	Serum	qRT-PCR	U6	HCC	30	HC	30	NA	70.0	86.7	0.824
Ning et al. (Ning et al., 2019)	2019	China	miR-99a	Down	Serum	qRT-PCR	U6	HCC	30	HC	30	NA	73.3	83.3	0.799
Ning et al. (Ning et al., 2019)	2019	China	miR-155, 96, 99a	NA	Serum	qRT-PCR	U6	HCC	30	HC	30	NA	76.7	96.7	0.931
Chen et al. (Chen and Wang, 2019)	2019	China	miR-195	Down	Serum	qRT-PCR	miR-16	HCC	120	HC	118	1.685	76.7	77.0	0.862
Zeng et al. (Zeng et al., 2020)	2020	China	miR-22	Down	Serum	qRT-PCR	U6	HCC	108	HC	67	1.352	89.3	68.9	0.866
Cao et al. (Cao and Wang, 2020)	2020	China	miR-768-3p	Down	Serum	qRT-PCR	cel-miR-39-3p	HBV-HCC	110	HC	60	0.809	87.27	80	0.908
Wu et al. (Wu et al., 2020)	2020	China	miR-199a	Down	Serum	qRT-PCR	cel-miR-54-5p	HCC	48	HC	50	1.02	91.7	62.0	0.808
Zhao et al. (Zhao et al., 2021)	2021	China	miR-324-3p	Up	Serum	qRT-PCR	cel-miR-39-3p	HBV-HCC	96	HC	76	1.608	77.08	93.42	0.926
Chen et al. (Chen et al., 2021)	2021	China	miR-497	Down	Serum	qRT-PCR	cel-miR-39	HCC	50	HC	50	NA	74.0	66.0	0.726
Chen et al. (Chen et al., 2021)	2021	China	miR-1246	Up	Serum	qRT-PCR	cel-miR-39	HCC	50	HC	50	NA	82.0	80.0	0.865
Chen et al. (Chen et al., 2021)	2021	China	miR-497, 1,246	NA	Serum	qRT-PCR	cel-miR-39	HCC	50	HC	50	NA	94.0	70.0	0.911
Wu et al. (Wu et al., 2022)	2022	China	miR-126	Down	Plasma	qRT-PCR	hsamiR-16-5p	HCC	38	HC	20	2.082	81.6	65	0.751
Wu et al. (Wu et al., 2022)	2022	China	miR-222	Up	Plasma	qRT-PCR	hsamiR-16-5p	HCC	38	HC	20	2.207	55.3	90	0.686
Wu et al. (Wu et al., 2022)	2022	China	miR-206	Up	Plasma	qRT-PCR	hsamiR-16-5p	HCC	38	HC	20	1.315	51.9	90	0.713
Wu et al. (Wu et al., 2022)	2022	China	miR-126, 206	NA	Plasma	qRT-PCR	hsamiR-16-5p	HCC	38	HC	20	NA	81.6	85	0.887
Fang et al. (Fang et al., 2022)	2022	China	miR-16	Up	Serum	qRT-PCR	hsamiR-21-5p	HCC	100	HC	100	NA	91	58	0.798
Shaheen et al. (Shaheen et al., 2018)	2018	Egypt	miR-150	Down	Serum	qRT-PCR	cel-mir-39	HCV-HCC	40	HC	40	0.674	60	70	0.638
Elmougy et al. (Elmougy et al., 2019)	2019	Egypt	miR-223	Down	Serum	qRT-PCR	SNORD68	HCV-HCC	40	HC	40	1.59	77.5	80.0	0.857
Elmougy et al. (Elmougy et al., 2019)	2019	Egypt	miR-19a	Up	Serum	qRT-PCR	SNORD68	HCV-HCC	40	HC	40	0.65	70	77.5	0.726
Mahdy et al. (El Mahdy et al., 2019)	2019	Egypt	miR-215	Down	Plasma	qRT-PCR	RNU6	HCC	60	HC	60	2.30	80	96.7	0.93
Aly et al. (Aly et al., 2020)	2020	Egypt	miR-143	Down	Serum	qRT-PCR	SNORD68	HCV-HCC	40	HC	40	0.43	62.5	72.5	0.702
Aly et al. (Aly et al., 2020)	2020	Egypt	miR-145	Down	Serum	qRT-PCR	SNORD68	HCV-HCC	40	HC	40	0.462	65	67.5	0.677
Elhendawy et al. (Elhendawy et al., 2020)	2020	Egypt	miR-142-5p	Up	Plasma	NGS	NA	HCC	20	HC	10	1.401	75.0	100.0	0.929
Elhendawy et al. (Elhendawy et al., 2020)	2020	Egypt	miR-191-5p	Down	Plasma	NGS	NA	HCC	20	HC	10	4.426	92.9	80.0	0.929
Elhendawy et al. (Elhendawy et al., 2020)	2020	Egypt	miR-22-3p	Down	Plasma	NGS	NA	HCC	20	HC	10	2.165	76.9	100.0	0.831
Elhendawy et al. (Elhendawy et al., 2020)	2020	Egypt	miR-126-5p	Down	Plasma	NGS	NA	HCC	20	HC	10	2.315	83.0	100.0	0.967
Wahb et al. (Wahb et al., 2021)	2021	Egypt	miR-9-3p	Down	Serum	qRT-PCR	U6 snRNA	HCV-HCC	35	HC	32	1.01	91.43	87.50	N/A
Elfert et al. (Elfert et al., 2022)	2022	Egypt	miR-122	Up	Serum	qRT-PCR	SNORD68	HCV-HCC	90	HC	60	6.55	100	84.1	0.95
Elfert et al. (Elfert et al., 2022)	2022	Egypt	miR-483	Up	Serum	qRT-PCR	SNORD68	HCV-HCC	90	HC	60	2.43	100	82.3	0.986
Elfert et al. (Elfert et al., 2022)	2022	Egypt	miR-335	Down	Serum	qRT-PCR	SNORD68	HCV-HCC	90	HC	60	0.49	100	79.8	0.908
Youssef et al. (Youssef et al., 2022)	2022	Egypt	miR-326	Up	Plasma	qRT-PCR	RNU6B	HCC	70	HC	20	1.165	97.1	52.0	0.784
Youssef et al. (Youssef et al., 2022)	2022	Egypt	miR-511	Down	Plasma	qRT-PCR	RNU6B	HCC	70	HC	20	2.063	71.4	60.0	0.654
Youssef et al. (Youssef et al., 2022)	2022	Egypt	miR-424	Down	Plasma	qRT-PCR	RNU6B	HCC	70	HC	20	2.462	82.9	48.0	0.559
Yousuf et al. (Yousuf et al., 2022)	2022	India	miR-221	Down	Serum	qRT-PCR	U6	HCC	33	HC	33	1.626	77.14	80.77	0.786
Yousuf et al. (Yousuf et al., 2022)	2022	India	miR-222	Down	Serum	qRT-PCR	U6	HCC	33	HC	33	0.609	86.96	68.75	0.758
Loosen et al. (Loosen et al., 2021)	2021	Germany	miR-107	Up	Serum	qPCR	NA	HCC	45	HC	18	2.63	55.6	100	0.679
Moshiri et al. (Moshiri et al., 2018)	2018	Italy	miR-101-3p	Up	Plasma	ddPCR	NA	HCC	29	HC	25	NA	71.4	58.8	0.71
Moshiri et al. (Moshiri et al., 2018)	2018	Italy	miR-1246	Up	Plasma	ddPCR	NA	HCC	29	HC	25	NA	57.1	78.6	0.83
Moshiri et al. (Moshiri et al., 2018)	2018	Italy	miR-106b-3p	Up	Plasma	ddPCR	NA	HCC	29	HC	25	NA	87.0	83.3	0.95
Moshiri et al. (Moshiri et al., 2018)	2018	Italy	miR-101-3p, 1246,106b-3p	NA	Plasma	ddPCR	NA	HCC	29	HC	25	NA	100.0	100.0	1.00

Note: HC: healthy control; HCC: hepatocellular carcinoma; HCV-HCC: hepatitis C virus related hepatocellular carcinoma; HBV-HCC: hepatitis B virus related hepatocellular carcinoma; NA: not available; NGS: next-generation sequence; qRT-PCR: quantitative real-time polymerase chain reaction; qPCR: quantitative polymerase chain reaction; ddPCR: droplet digital polymerase chain reaction.

**FIGURE 2:**
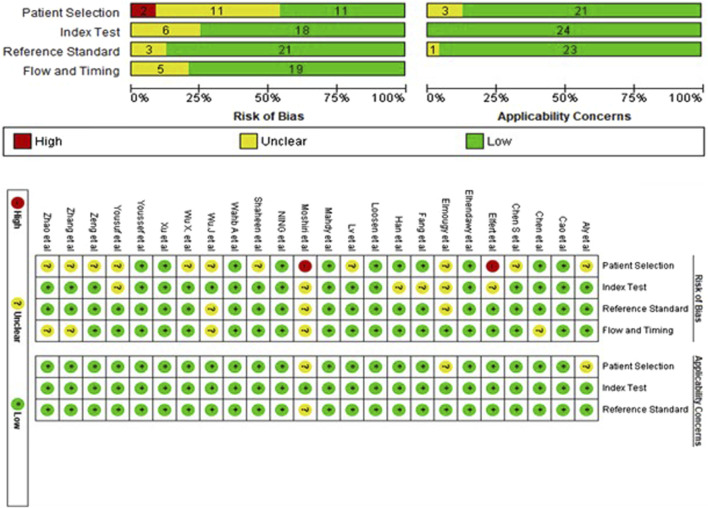
The risk of bias assessment of included studies using the QUADAS-2 tool evaluates the quality of diagnostic accuracy studies across four domains: patient selection, index test, reference standard, and flow and timing.

The assessment of the 24 studies’ quality was conducted using the QUADAS-2 tool. Given the significance of patient selection in experimental integrity, the data utilized in this meta-analysis predominantly originated from validated groups. Overall, the included studies exhibited satisfactory and qualifying methodological standards. Figure 2 provides a detailed breakdown of the quality assessment criteria.

### Overall diagnostic accuracy of circulating miRNAs in diagnosing HCC

The presence of a threshold effect of heterogeneity was evaluated using both the Spearman correlation coefficient and the SROC curve. The findings from the Spearman correlation coefficient (with a rho value of 0.15 and a *p*-value of 0.28) and the absence of the characteristic “shoulder-arm” shape in the SROC curve indicated that there is no evidence of a threshold effect of heterogeneity. In addition, the I^2^ values for sensitivity, specificity, PLR, NLR, and DOR were 82.2%, 74.28%, 65.07%, 78.88%, and 100%, respectively. With I^2^ results exceeding 50% and *p*-values for all parameters below 0.001, it strongly suggests the presence of substantial non-threshold effect heterogeneity in this study. Therefore, a random-effects model was employed for the meta-analysis.

**FIGURE 3:**
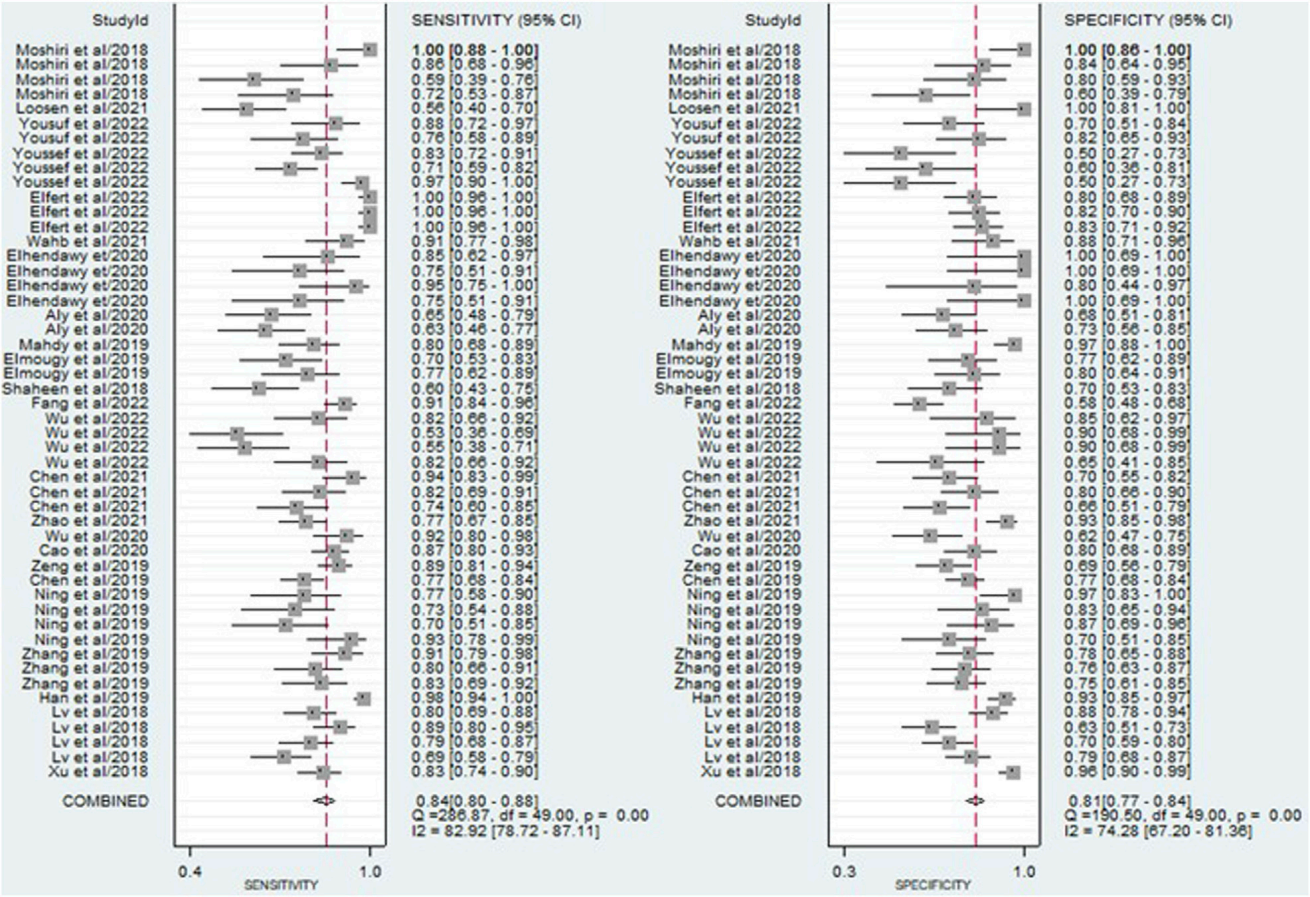
A forest plot illustrating the pooled sensitivity and specificity estimates of miRNAs for diagnosing HCC across multiple studies, providing an overview of the diagnostic performance of miRNAs.

**FIGURE 4:**
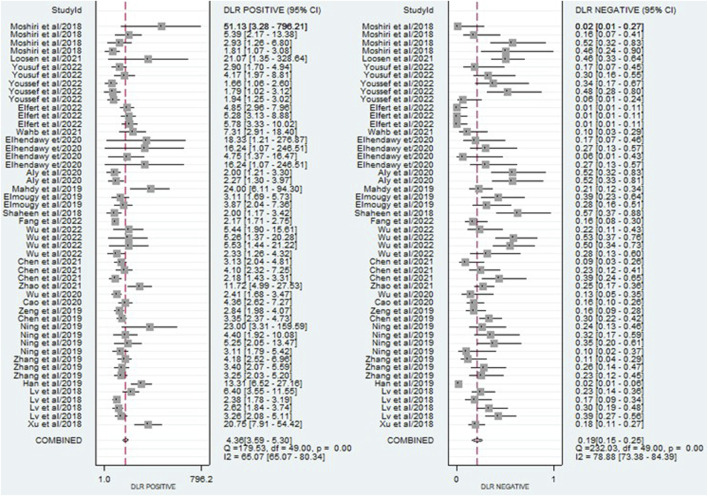
A forest plot showing the pooled PLR and NLR estimates of miRNAs for diagnosing HCC, providing insights into the diagnostic utility of miRNAs.

**FIGURE 5:**
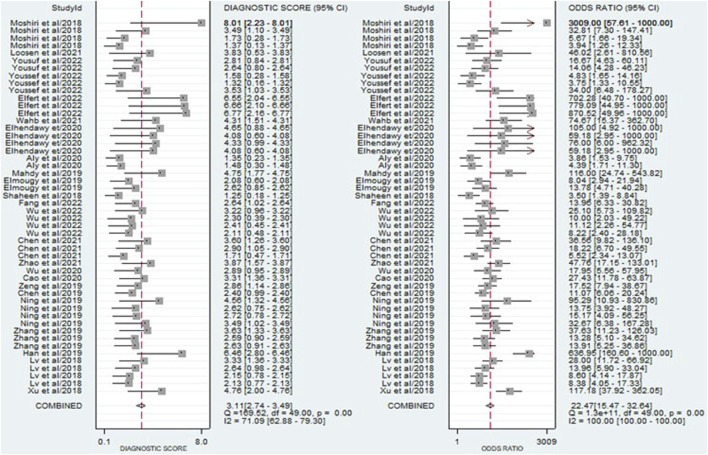
A forest plot illustrating the pooled DOR estimates of miRNAs for diagnosing HCC, offering a comprehensive evaluation of the overall diagnostic performance of miRNAs.

**FIGURE 6:**
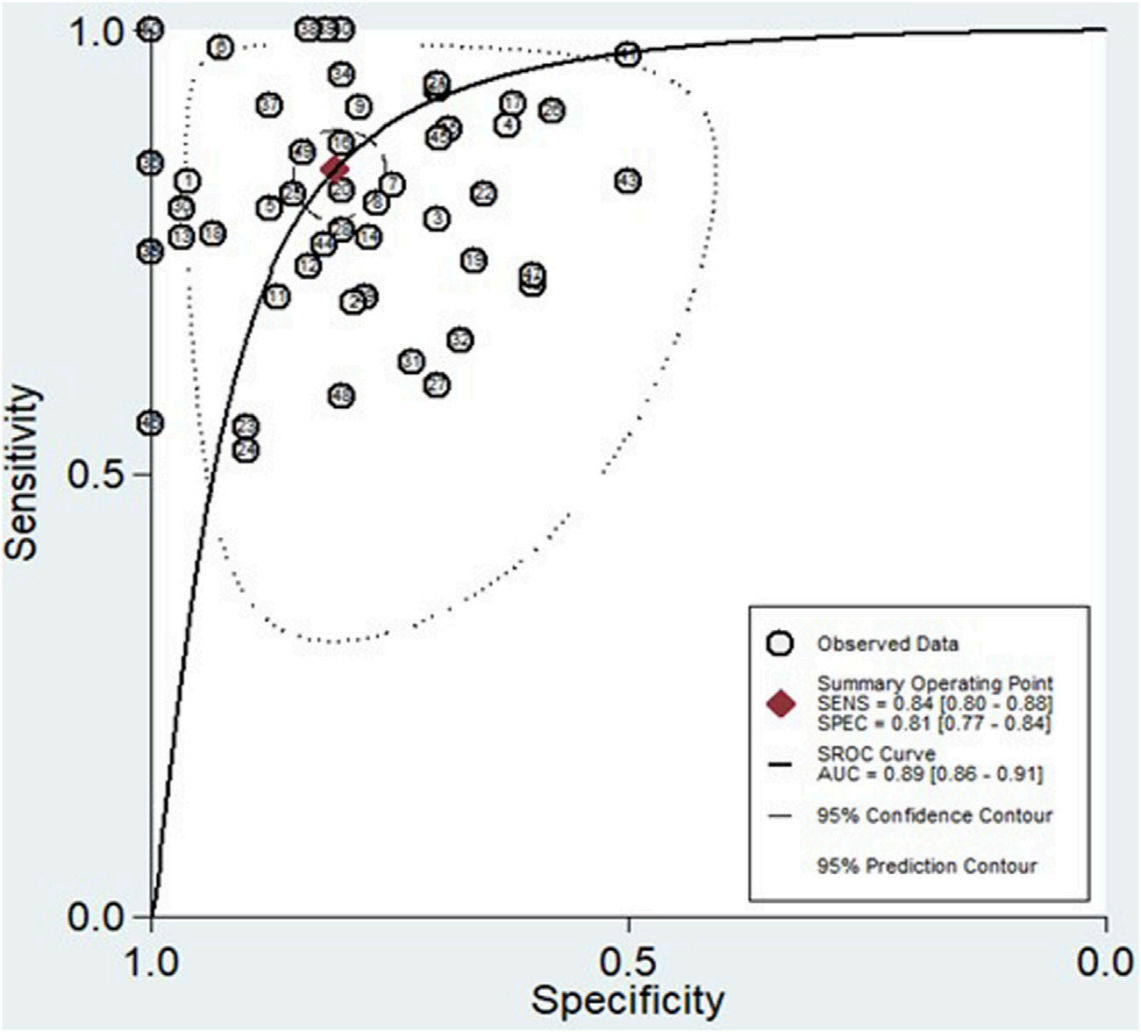
The SROC curve, accompanied by the 95% confidence contour and 95% prediction contour, provides a graphical representation of the overall diagnostic accuracy of miRNAs in distinguishing HCC.

The findings revealed that circulating miRNAs demonstrated strong diagnostic potential for detecting HCC. The combined sensitivity and specificity were 0.84 (95% CI: 0.80–0.88) and 0.81 (95% CI: 0.77–0.84), respectively (Figure 3). Additionally, the pooled PLR and NLR were 4.36 (95% CI: 3.59–5.30) and 0.19 (95% CI: 0.15–0.25), respectively (Figure 4). Furthermore, the DOR was 22.47 (95% CI: 14.47–32.64) (Figure 5). In assessing diagnostic accuracy, the SROC curve was generated, resulting in an AUC of 0.89 (95% CI: 0.86–0.91) (Figure 6). These results indicate that circulating miRNAs exhibit high diagnostic accuracy in identifying HCC, as an AUC greater than 0.7 is indicative of a strong predictive capability.

### Clinical applicability of miRNAs for HCC diagnosis

**FIGURE 7:**
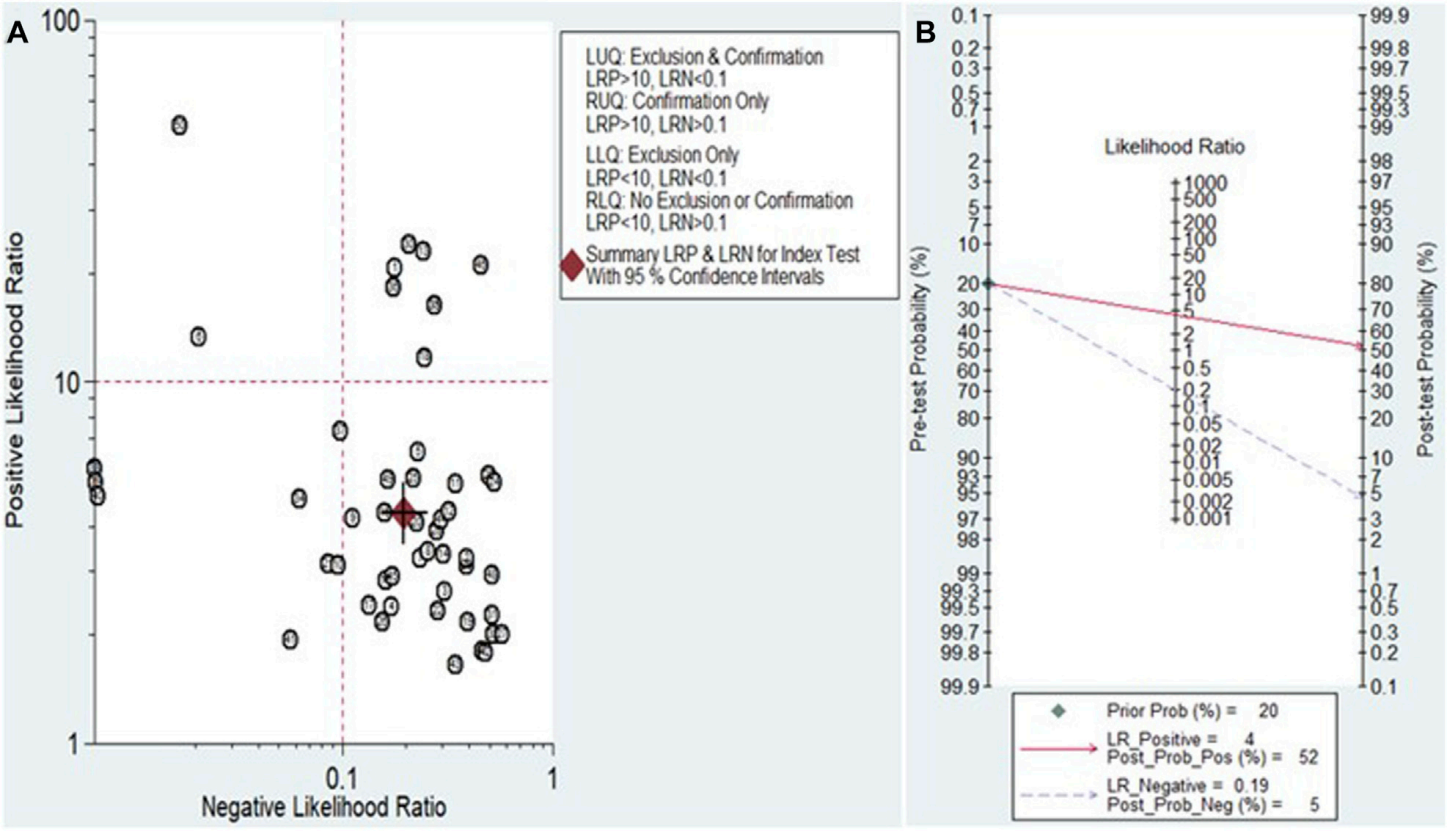
Assessment of the clinical applicability of miRNAs for the diagnosis of HCC. **(A)** Likelihood ratio scattergram visually represents the relationship between pre-test and post-test probabilities of HCC diagnosis based on miRNA test results. **(B)** Fagan’s nomogram, a graphical tool for estimating the post-test probability of HCC given a specific pre-test probability and miRNA test result.

The Fagan nomogram and likelihood ratio scattergram were used to evaluate the clinical value of miRNAs in HCC diagnosis. The Fagan’s nomogram showed encouraging outcomes, revealing post-test probabilities of 0.52 and 0.5 for PLR and NLR respectively, under a pre-test probability set at 20% (Figure 7B). Furthermore, a scattergram depicting the likelihood ratios (PLR and NLR) was generated to assess the clinical utility of miRNAs in HCC diagnosis. The findings indicated that the studies conducted by Han et al. (miR-148a) (Han et al., 2019) and Moshiri et al. (miR-101-3p, miR-1246, and miR-106b-3p) (Moshiri et al., 2018) laid over on a left upper quadrant (PLR >10 and an NLR <0.1) (Figure 7A).

### Subgroup analysis, meta-regression and sensitivity analysis

Because significant heterogeneity (I^2^ > 50% and *p* < 0.05) was observed across all diagnostic performance parameters (including sensitivity, specificity, PLR, NLR, DOR, and AUC), meta-regression and subgroup analyses were performed. These analyses aimed to investigate the sources of heterogeneity among the studies by exploring various study characteristics. These characteristics encompass ethnicity, biological specimen, regulation mode, miRNA profiling, method of identification, internal reference, sample size, cut-off value establishment, and the classification of HCC patients.

In the subgroup analysis, plasma-derived miRNAs demonstrated superior diagnostic performance for HCC compared to serum-derived miRNAs. Plasma-derived miRNAs showed higher sensitivity (0.84, 95% CI: 0.75–0.90), specificity (0.86, 95% CI: 0.76–0.92), PLR (6.0, 95% CI: 3.3–11.0), NLR (0.19, 95% CI: 0.12–0.31), DOR (32, 95% CI: 13–79), and AUC (0.92, 95% CI: 0.89–0.94) compared to serum-derived miRNAs (sensitivity: 0.85, 95% CI: 0.80–0.89; specificity: 0.79, 95% CI: 0.75–0.82; PLR: 4.0, 95% CI: 3.3–4.8; NLR: 0.19, 95% CI: 0.14–0.26; DOR: 20, 95% CI: 14–30; AUC: 0.87, 95% CI: 0.84–0.90). Furthermore, within the subgroups categorized by miRNA profiling, miRNA clusters exhibited higher accuracy in HCC detection compared to individual single miRNAs. MiRNA clusters displayed a sensitivity of 0.88 (95% CI: 0.79–0.94), specificity of 0.88 (95% CI: 0.76–0.94), PLR of 7.1 (95% CI: 3.5–14.6), NLR of 0.13 (95% CI: 0.07–0.24), DOR of 53 (95% CI: 19–144), and an AUC of 0.94 (95% CI: 0.92–0.96).

Regarding sample size, miRNAs demonstrated the highest overall diagnostic accuracy when the sample size was ≥100, with a sensitivity of 0.90 (95% CI: 0.83–0.94), specificity of 0.81 (95% CI: 0.75–0.85), PLR of 4.7 (95% CI: 3.5–6.2), NLR of 0.13 (95% CI: 0.08–0.21), DOR of 37 (95% CI: 19–71), and an AUC of 0.91 (95% CI: 0.88–0.93) compared to cases with a sample size <100. Additionally, miRNAs proved to be highly effective in diagnosing undefined HCC in comparison to HCV-related HCC. For undefined HCC, the diagnostic values were as follows: sensitivity of 0.83 (95% CI: 0.78–0.86), specificity of 0.80 (95% CI: 0.75–0.84), PLR of 4.2 (95% CI: 3.3–5.3), NLR of 0.22 (95% CI: 0.17–0.27), DOR of 19 (95% CI: 14–28), and an AUC of 0.88 (95% CI: 0.85–0.91) (Table 2).

**TABLE 2:** Subgroup analysis of the diagnostic accuracy of miRNAs in HCC.

Subgroup	No of studies	Sen (95% CI)	Spe (95% CI)	PLR (95% CI)	NLR (95% CI)	DOR (95% CI)	AUC (95% CI)
Ethnicity							
Asian	28	0.83 (0.79, 0.87)	0.80 (0.75, 0.84)	4.1 (3.3, 5.2)	0.21 (0.17, 0.27)	20 (14, 28)	0.88 (0.85, 0.91)
Non-Asian	22	0.87 (0.78, 0.93)	0.82 (0.75, 0.88)	5.0 (3.3, 7.4)	0.15 (0.08, 0.28)	33 (15, 87)	0.91 (0.88, 0.93)
Specimen							
Serum	33	0.85 (0.80, 0.89)	0.79 (0.75, 0.82)	4.0 (3.3, 4.8)	0.19 (0.14, 0.26)	20 (14, 30)	0.87 (0.84, 0.90)
Plasma	17	0.84 (0.75, 0.90)	0.86 (0.76, 0.92)	6.0 (3.3, 11.0)	0.19 (0.12, 0.31)	32 (13, 79)	0.92 (0.89, 0.94)
Regulation mode							
Up	20	0.83 (0.74, 0.90)	0.80 (0.74, 0.85)	4.2 (3.2, 5.4)	0.21 (0.13, 0.32)	20 (12, 34)	0.88 (0.85, 0.91)
Down	24	0.84 (0.78, 0.88)	0.79 (0.73, 0.84)	4.0 (3.0, 5.4)	0.21 (0.15, 0.28)	20 (11, 34)	0.88 (0.84, 0.90)
miRNAs profile							
Single	44	0.84 (0.79, 0.88)	0.80 (0.75, 0.83)	4.1 (3.4, 5.0)	0.21 (0.16, 0.27)	20 (13, 29)	0.88 (0.85, 0.91)
Combination	6	0.88 (0.79, 0.94)	0.88 (0.76, 0.94)	7.1 (3.5, 14.6)	0.13 (0.07, 0.24)	53 (19, 144)	0.94 (0.92, 0.96)
References							
U6	15	0.82 (0.78, 0.86)	0.80 (0.74, 0.85)	4.1 (3.2, 5.4)	0.22 (0.18, 0.27)	19 (13, 27)	0.88 (0.85, 0.90)
Others	35	0.86 (0.79, 0.90)	0.81 (0.76, 0.85)	7.5 (3.5, 5.9)	0.18 (0.12, 0.26)	25 (15, 44)	0.89 (0.86, 0.92)
Methods							
qRT-PCR	41	0.85 (0.80, 0.89)	0.79 (0.75, 0.82)	4.0 (3.3, 4.8)	0.19 (0.14, 0.25)	21 (14, 31)	0.88 (0.85, 0.91)
Others	9	0.81 (0.68, 0.90)	0.95 (0.76, 0.99)	14.4 (2.9, 80.8)	0.20 (0.11, 0.36)	77 (11, 538)	0.93 (0.91, 0.95)
Sample size							
<100	30	0.79 (0.74, 0.84)	0.81 (0.75, 0.85)	4.1 (3.1, 5.3)	0.26 (0.20, 0.33)	16 (10, 24)	0.87 (0.83, 0.89)
≥100	20	0.90 (0.83, 0.94)	0.81 (0.75, 0.85)	4.7 (3.5, 6.2)	0.13 (0.08, 0.21)	37 (19, 71)	0.91 (0.88, 0.93)
Cut-off value							
Reported	32	0.85 (0.79, 0.90)	0.82 (0.77, 0.86)	4.7 (3.6, 6.2)	0.18 (0.12, 0.26)	26 (15, 44)	0.90 (0.87, 0.92)
Not reported	18	0.90 (0.83, 0.94)	0.81 (0.75, 0.85)	4.7 (3.5, 6.2)	0.13 (0.08, 0.21)	37 (19, 71)	0.91 (0.88, 0.93)
Participants							
Undefined HCC	38	0.83 (0.78, 0.86)	0.80 (0.75, 0.84)	4.2 (3.3, 5.3)	0.22 (0.17, 0.27)	19 (14, 28)	0.88 (0.85, 0.91)
HCV related HCC	9	0.93 (0.71, 0.99)	0.78 (0.73, 0.82)	4.2 (3.1, 5.6)	0.09 (0.02, 0.46)	48 (7, 317)	0.81 (0.78, 0.85)
HBV related HCC	3	-	-	-	-	-	-

**FIGURE 8:**
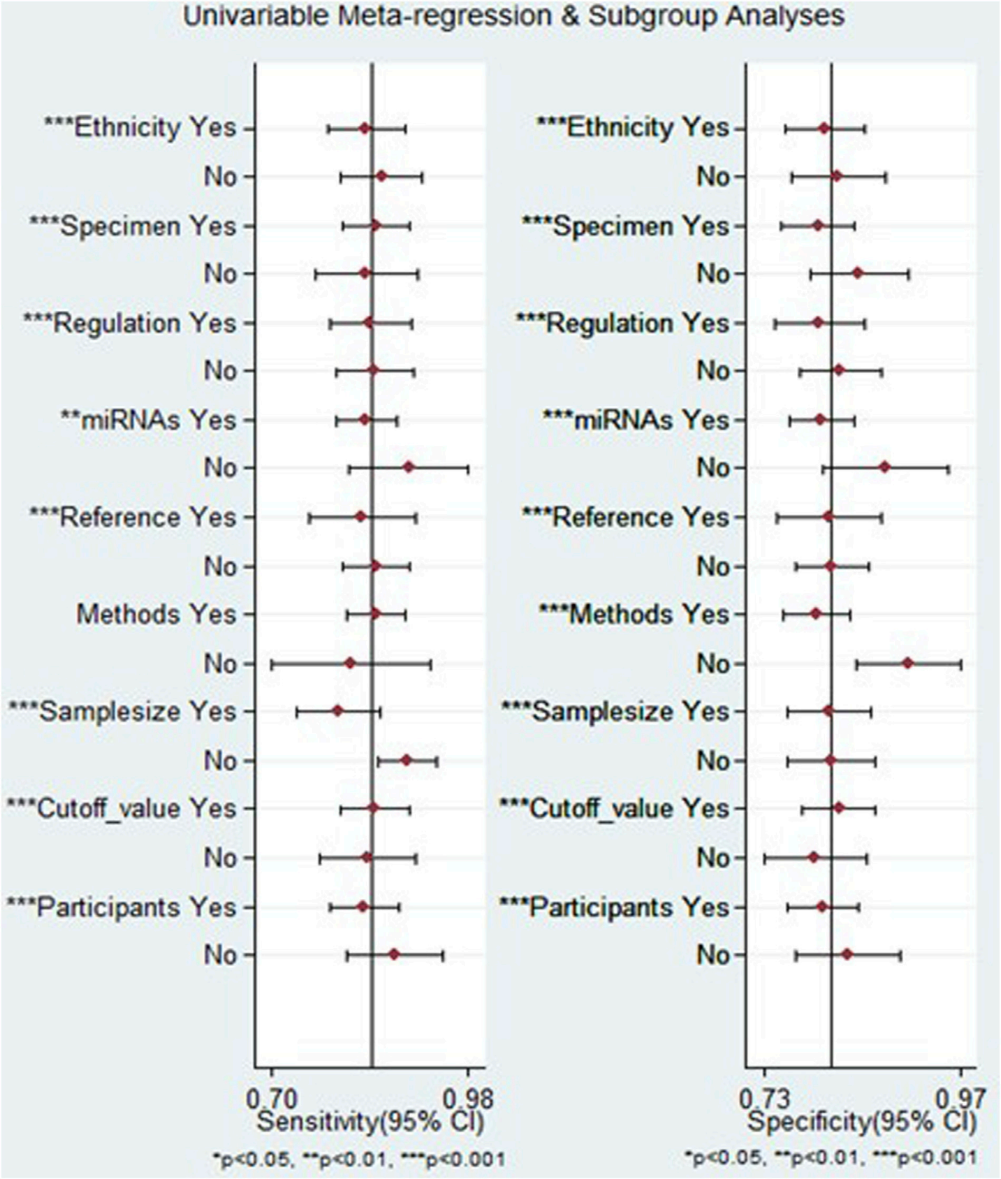
The results of meta-regression analysis examining the relationship between sensitivity and specificity of miRNA-based diagnostics for HCC, exploring potential sources of heterogeneity across included studies.

The outcomes of the meta-regression analysis revealed that for both sensitivity and specificity, sources of heterogeneity included ethnicity, biological specimen, regulation mode, miRNA profiling, internal reference, sample size, cut-off value determination, and the classification of HCC patients (*p* < 0.05). In contrast, the method of identification was identified as a source of heterogeneity specifically for specificity (*p* < 0.05) (Figure 8).

**FIGURE 9:**
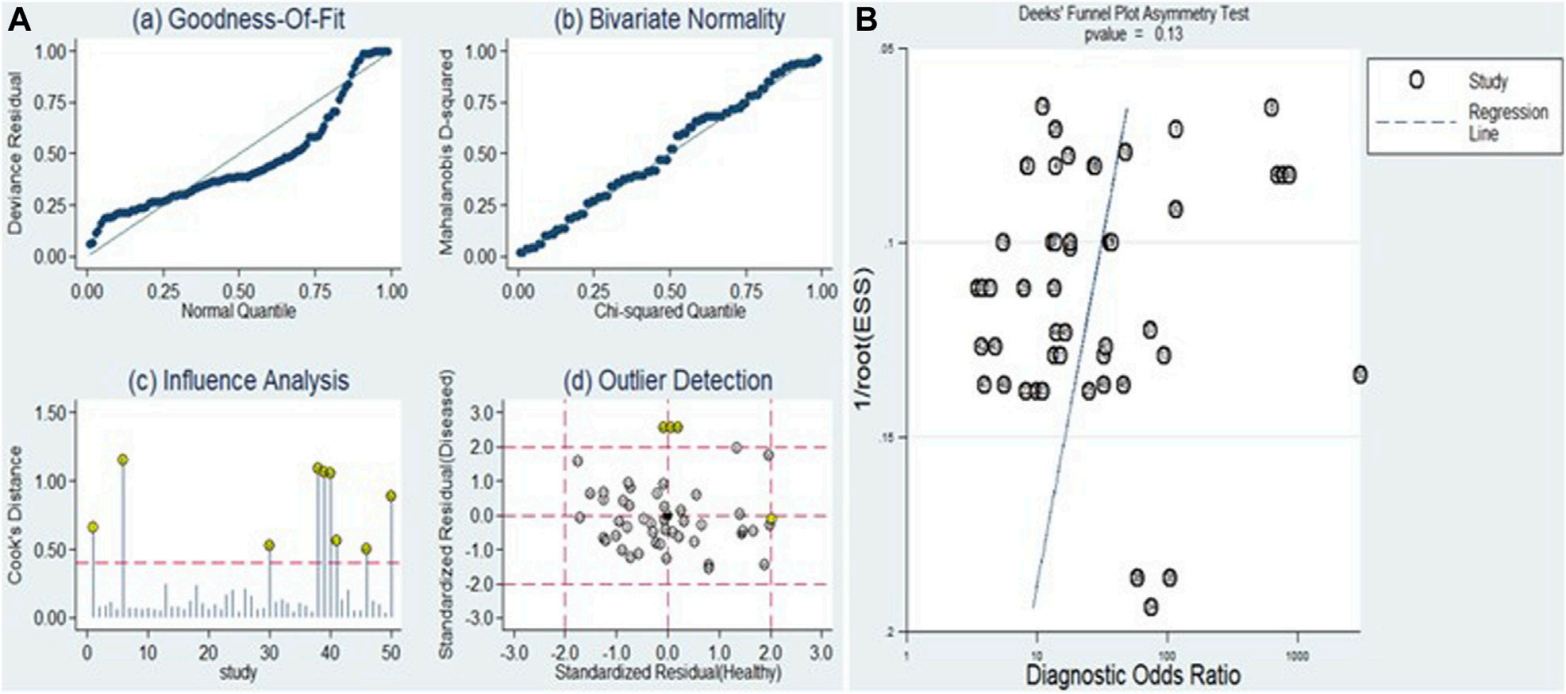
Sensitivity analysis and assessment of publication bias. **(A)** Sensitivity analysis evaluates the robustness of pooled estimates by examining the impact of various factors or assumptions on the overall findings. **(B)** Deek’s funnel plot is used to detect publication bias by examining the relationship between study size and effect size estimates.

The sensitivity analysis can be observed in Figure 9A. Examination of goodness-of-fit and bivariate normality indicated the strength and reliability of the bivariate mixed-effects model for conducting meta-analysis (Figure 9A (a and b)). Moreover, the identification of outliers pointed to potential sources of heterogeneity in the form of two studies conducted by Xu et al. (miR-125b) and Elfert et al. (miR-122, miR-483, and miR-335) (Figure 9A (d)). Upon removing these outliers, we observed no substantial alterations in the overall sensitivity (0.82, 95% CI: 0.78–0.85), specificity (0.80, 95% CI: 0.76–0.84), PLR (4.1, 95% CI: 3.3–4.9), NLR (0.23, 95% CI: 0.19–0.28), DOR (18, 95% CI: 13–24), and AUC (0.88, 95% CI: 0.84–0.90). This suggests that the sensitivity of the studies included was consistently low, and the results became more resilient and trustworthy.

### Publication bias

The Deeks’ funnel plot asymmetry test was executed (Figure 9B) to evaluate the presence of publication bias. The resulting *p*-value of 0.13 indicates that there was no evidence of publication bias within the included studies.

## Discussion

Hepatocellular carcinoma is a widespread cancer within the digestive system and stands as a primary contributor to global cancer-related fatalities. Challenges persist in detecting the disease at its early stages, coupled with the substantial risk of tumor recurrence and metastasis following surgery. Consequently, HCC continues to pose a significant threat to both human health and survival (Villanueva, 2019). The disease begins gradually and progresses slowly, resulting in atypical early clinical signs. A considerable number of individuals with HCC receive inadequate diagnoses until the disease reaches its mid-to-late stages, leading to the loss of the optimal treatment period (Yang and Heimbach, 2020). Current diagnostic methods for HCC, such as imaging techniques and serum biomarkers, often have limitations in terms of accuracy and invasiveness (Bialecki and Di Bisceglie, 2005). Therefore, there is an urgent need for the identification of non-invasive biomarkers for the detection of HCC.

MiRNAs exhibit resilience in circulation through the formation of inclusion bodies and exosomes, allowing for their extraction and examination from biological fluids like serum and stool samples (Valihrach et al., 2020). Disruption in their expression is a common occurrence in cancer (Shaker et al., 2022). This dysregulation leads to a distinct expression profile that is advantageous for early cancer detection. Specifically, miRNAs linked to tumor growth are often overexpressed, while suppressors are frequently under-expressed (Shaker et al., 2021). In recent years, the role of miRNAs in HCC has garnered increasing attention, attributed to their involvement in key signaling pathways implicated in hepatocarcinogenesis (Ruiz-Manriquez et al., 2022). These miRNAs play crucial roles in modulating various cellular processes integral to HCC pathogenesis, including cell proliferation, apoptosis, migration, invasion, angiogenesis, and drug resistance (Xu X. et al., 2018). Circulating miRNAs, protected from RNases, display notable stability (Cui et al., 2019). This stability persists even in challenging conditions such as boiling, extended storage, numerous freeze–thaw cycles, and exposure to extremely low or high pH (Drees and Pegtel, 2020). These characteristics suggest that miRNAs are widely distributed and hold promise for effective early detection of HCC (Shen et al., 2013). Consequently, this systematic review and meta-analysis aimed to assess the diagnostic accuracy of circulating miRNAs in detecting HCC.

In the present study, a total of 24 articles, involving 1,668 HCC patients and 1,236 healthy controls were included. The results indicate that the pooled sensitivity, specificity, PLR, NLR, DOR, and AUC of miRNAs in the diagnosis of HCC were 0.84 (95% CI: 0.80–0.88), 0.81 (95% CI: 0.77–0.84), 4.36 (95% CI: 3.59–5.30), 0.19 (95% CI: 0.15–0.25), 22.47 (95% CI: 14.47–32.64), and 0.89 (95% CI: 0.86–0.91), respectively. The PLR value of 4.3 suggests that the likelihood of a positive miRNA determination in patients with HCC is approximately 4.3 times higher compared to healthy controls. On the other hand, the NLR of 0.19 indicates that cases with negative test results have about a 19% chance of developing HCC. The DOR serves as an index indicating the discriminatory performance of a test (Glas et al., 2003), and a DOR value exceeding 1 signifies a superior diagnostic test. With a DOR of 22.47, the miRNA exhibits a substantial ability to effectively differentiate between HCC patients and healthy controls. Additionally, the AUC value is a valuable indicator for evaluating a system. An ideal test demonstrating perfect discrimination achieves an AUC of 1.0 (Nahm, 2022). As the AUC value approaches 1.0, the overall effectiveness of the test increases. In this study, miRNA emerges as a best tool for screening HCC patients compared to healthy controls, boasting an AUC value of 0.89, which is in close proximity to 1.0. This suggests that miRNAs possess a relatively high capability to distinguish between HCC patients and healthy controls.

In a meta-analysis conducted by Jiang et al., the effectiveness of miRNAs as diagnostic markers for HCC was explored. The reported diagnostic values of miRNAs are as follows: sensitivity of 0.84 (95% CI, 0.79–0.88), specificity of 0.87 (95% CI, 0.83–0.90), a DOR of 36 (95% CI: 20–64), and an AUC of 0.92 (95% CI, 0.90–0.94). These findings highlight the potential diagnostic value of miRNAs for individuals with HCC (Jiang et al., 2019). Discrepancies in certain pooled diagnostic values between our results and those of Jiang et al. could be attributed to variations in diagnostic criteria, demographic differences, adjustments in sample size, and differences in study design. Additionally, discrepancies may arise from variations in miRNA expression patterns, biological diversity, and temporal changes in the characteristics of HCC, all contributing to differences in the reported pooled diagnostic accuracy.

Due to the presence of heterogeneity among the included studies, subgroup analysis and meta-regression analysis were conducted to investigate potential confounding factors. Subgroup analysis indicated that plasma-derived miRNAs exhibited better diagnostic performance (AUC: 0.92) for HCC compared to serum-derived miRNAs (AUC: 0.87). Our findings align with those reported in a meta-analysis conducted by Wu et al. (Wu et al., 2023). Differences in miRNA concentration between serum and plasma can be attributed to various factors, including platelet contamination along with red and white blood cells (Wang et al., 2012), hemolysis (Kirschner et al., 2011), and the presence of qPCR inhibitors (Kaudewitz et al., 2013). Additionally, beyond sample contamination by miRNA from red blood cell rupture, the miRNA profile in serum and plasma may be influenced by platelet content or activation. Activated platelets are known to release miRNAs incorporated into microparticles or the effector protein Argonaute 2 (Ago2) (Laffont et al., 2013). In serum, platelet-derived miRNAs may be released during the coagulation process, and in the case of plasma, there might be residual contamination even after careful serial centrifugation to deplete platelet content (Binderup et al., 2018).

Furthermore, miRNA panels (AUC: 0.94) demonstrated superior accuracy in detecting HCC compared to single miRNAs (AUC: 0.88). This heightened accuracy could be attributed to the involvement of multiple gene mutations and epigenetic genetic abnormalities in the development of HCC (Stavast and Erkeland, 2019). Consequently, miRNA panels may emerge as more suitable diagnostic biomarkers for HCC, reflecting a future trend in development. Interestingly, our results revealed a significant difference in diagnostic ability between groups with a sample size of ≥100 (AUC: 0.91) and <100 (AUC: 0.87). Thus, validating these findings will necessitate large sample sizes and extensive studies.

The paramount value of biomarkers lies in their contribution to clinical decision-making. Likelihood ratios (Rubinstein et al., 2018) serve as valuable tools for clinicians, offering insights into the likelihood that a patient with a positive or negative test actually has HCC or not. In this study, we summarized PLR and NLR to assess the clinical applicability of miRNAs for diagnosing HCC. A PLR >10 and NLR <0.1 indicate high diagnostic accuracy. Our findings highlight that specific miRNA panels, including miR-101-3p, miR-1246, and miR-106b-3p from Moshiri et al.’s (Moshiri et al., 2018) study, and miRNA-148a from Han et al.’s (Han et al., 2019) study, exhibit high diagnostic accuracy and clinical applicability. These miRNAs exert profound effects on HCC pathogenesis, offering intricate correlations and multifaceted functions. MiRNA-148a emerges as a potent tumor suppressor within HCC, orchestrating anti-tumor activities such as inhibiting cell proliferation, inducing apoptosis, and impeding tumor growth (Babu and Muckenthaler, 2019). Its ability to counteract HCC metastasis through the inhibition of the epithelial-mesenchymal transition process and the suppression of the Wnt/β-catenin signaling pathway underscores its pivotal role in mitigating disease progression (Zhang et al., 2014). Similarly, miR-101-3p functions as a tumor suppressor, exerting inhibitory effects on HCC proliferation, metastasis, and stemness properties through the regulation of oncogenic pathways (Su et al., 2009). In contrast, miR-1246 and miR-106b-3p demonstrate oncogenic potential in HCC, promoting tumor progression by enhancing cell proliferation, migration, invasion, and metastasis (Yau et al., 2013; Chuma et al., 2019). Additionally, Fagan’s nomogram reveals promising outcomes, with post-test probabilities of 0.52 and 0.5 for PLR and NLR, respectively, when the pre-test probability was set at 20%. This result suggests that when samples test positive for the presence of miRNAs, patients have a 52% probability of developing HCC. In contrast, the post-test probability of disease is reduced to 5% when the samples test negative for miRNAs. Consequently, miRNAs exhibit a certain diagnostic potential in distinguishing patients with HCC from healthy controls, making them a suitable screening method for HCC.

This meta-analysis has several strengths. It highlights those circulating miRNAs exhibit high diagnostic value in distinguishing healthy controls from patients with HCC, contributing valuable insights for the development of biomarkers in HCC diagnosis. Moreover, the inclusion of studies with high methodological quality minimizes the risk of bias, and the analysis encompasses a more comprehensive list of miRNAs. The incorporation of subgroup analysis and meta-regression analysis addresses the observed heterogeneity in the studies. However, certain limitations exist. Firstly, due to constraints in sample sizes and data within the included studies, the diagnostic efficacy of miRNA in different clinical stages and metastasis of HCC has not been thoroughly analyzed. Second, variations in cutoff values of miRNAs among the studies could contribute to heterogeneity. Third, the lack of consensus on a unified internal reference may result in inconsistent results in miRNA relative quantitative analysis. Fifth, the representation of only some countries may limit the global applicability of miRNAs’ diagnostic performance for HCC. Sixth, the relatively small sample size in each study may affect statistical power. Seventh, the absence of a large number of similar miRNAs for pooling results prevents the identification of specific single miRNA or miRNA panels as the optimal diagnostic biomarkers for HCC. Therefore, while these findings offer valuable insights, caution is warranted in interpretation. The results should be further validated through well-designed studies with larger sample sizes in the future to enhance their reliability and generalizability.

In conclusion, our findings highlight the robust diagnostic potential of circulating miRNAs in detecting HCC. With a combined sensitivity of 0.84 and specificity of 0.81, along with a pooled PLR of 4.36 and a NLR of 0.19, the DOR stood at 22.47. Evaluation of diagnostic accuracy via the SROC curve yielded an AUC of 0.89. These compelling results affirm that circulating miRNAs demonstrate high diagnostic accuracy in identifying HCC, as an AUC exceeding 0.7 signifies a robust predictive capability. Moreover, miRNA panels, plasma-derived miRNAs, and miRNAs analyzed in studies with a large sample size (≥100) demonstrate heightened diagnostic potency in HCC diagnosis. However, to validate and strengthen our findings, a comprehensive collection of prospective studies and multi-center research is essential in the near future.
