# Supplementary material for: Circulating microRNAs as promising diagnostic biomarkers for hepatocellular carcinoma: a systematic review and meta-analysis
Source: Front Mol Biosci. 2024 May 14;11:1353547. doi: 10.3389/fmolb.2024.1353547 (PMC11130514; doi:10.3389/fmolb.2024.1353547)
Supplement: Supplementary file 1 [file Table1.docx]

| **SN** | **Database** | **Search terms** | **Articles** | **date** |
| --- | --- | --- | --- | --- |
| 1 | Scopus | (TITLE-ABS-KEY (“diagnos*”)) AND ((TITLE-ABS-KEY (“plasma microRNAs")) OR (TITLE-ABS-KEY ("plasma microRNA")) OR (TITLE-ABS-KEY ("plasma miR*")) OR (TITLE-ABS-KEY ("serum miRNAs")) OR (TITLE-ABS-KEY ("serum microRNAs")) OR (TITLE-ABS-KEY ("serum microRNA")) OR (TITLE-ABS-KEY ("serum miR*"))) AND ((TITLE-ABS-KEY ("hepatocellular carcinoma")) OR (TITLE-ABS-KEY (hcc))) | 173 | 30/07/2023 |
| 2 | Embase | 'Diagnos*':ti,ab,kw AND ('circulating mirnas':ti,ab,kw OR 'circulating micrornas':ti,ab,kw OR 'circulating microrna':ti,ab,kw OR 'circulating mir*':ti,ab,kw OR 'plasma mirnas':ti,ab,kw OR 'plasma micrornas':ti,ab,kw OR 'plasma microrna':ti,ab,kw OR 'plasma mir*':ti,ab,kw OR 'serum mirnas':ti,ab,kw OR 'serum micrornas':ti,ab,kw OR 'serum microrna':ti,ab,kw OR 'serum mir*':ti,ab,kw) AND ('hepatocellular carcinoma':ti,ab,kw OR 'hcc':ti,ab,kw) | 316 | 30/07/2023 |
| 3 | Pubmed | (((((((Circulating [Title/Abstract]) OR (plasma [Title/Abstract])) OR (serum [Title/Abstract])) AND (miRNAs [Title/Abstract])) OR (microRNAs [Title/Abstract])) OR (miR*[Title/Abstract])) AND (diagnos*[Title/Abstract])) AND ("hepatocellular carcinoma"[Title/Abstract]) | 657 | 30/07/2023 |
| 4 | ScienceDirect | ("miRNAs" OR "miRNA" OR "miR ") AND ("diagnosis") AND ("hepatocellular carcinoma" OR "HCC") | 78 | 30/07/2023 |
| 5 | Wiley online library | ("miRNAs" OR "miRNA" OR "miR") AND ("diagnosis") AND ("hepatocellular carcinoma" OR "HCC") | 35 | 30/07/2023 |
| 6 | Other sources | Utilize the titles that were discovered from the reference lists of articles chosen during electronic database searches. | 7 | 30/07/2023 |

**Searching strategy for circulating microRNAs as promising diagnostic biomarkers for hepatocellular carcinoma: A systematic review and meta-analysis**
